# StayBalanced: implementation of evidence-based fall prevention balance training for older adults—cluster randomized controlled and hybrid type 3 trial

**DOI:** 10.1186/s13063-021-05091-1

**Published:** 2021-02-26

**Authors:** Alexandra Halvarsson, Kirsti Skavberg Roaldsen, Per Nilsen, Ing-Mari Dohrn, Agneta Ståhle

**Affiliations:** 1 Division of Physiotherapy, Department of Neurobiology, Care Sciences and Society, Karolinska Institutet, 23 100, SE-141 83 Huddinge, Sweden; 2 Allied Health Professionals Function, Karolinska University Hospital, Stockholm, Sweden; 3 Department of Research, Sunnaas Rehabilitation Hospital, Bjørnemyrveien 11, NO-1453 Bjørnemyr, Norway; 4 Department of Physiotherapy, Faculty of Health Sciences, Oslo Metropolitan University, Box 4, St. Olavsplass, NO-0130 Oslo, Norway; 5 Department of Medical and Health Sciences, Linköping University, SE-581 83 Linköping, Sweden; 6 Department of Neurobiology, Care Sciences and Society, Aging Research Center, Karolinska Institutet, SE-171 77 Stockholm, Sweden

**Keywords:** Implementation, Older adults, Balance training, Transferring knowledge, Clinical practice

## Abstract

**Background:**

The StayBalanced programme has shown positive effects on fall prevention, balance control and fear of falling. Despite convincing evidence on the efficacy and effectiveness of balance training, there is a gap between research findings and what is provided in community-based and clinical health care settings. Therefore, transferring evidence-based balance training into clinical practice is needed.

**Methods:**

This project, designed as a hybrid type 3 trial, is a cluster-randomized study with a mixed-method design, carried out in primary health care settings. The aim is to investigate the effectiveness of two different strategies to facilitate the implementation of an intervention, the StayBalanced balance training programme, in primary health care, including evaluation of relative changes and maintenance in patient outcomes between intervention arms over 24 months. The StayBalanced programme will be launched through a website with information on the balance training and how to use it in clinical practice. One implementation strategy will include close facilitation, i.e. support and close follow-ups initiated by the researchers, in addition to access to the website. The other strategy simply includes access to the StayBalanced website. Outcome measures in the project consist of implementation outcomes, such as acceptability, feasibility, fidelity and sustainability of the StayBalanced programme. Outcomes at an individual level for older adults participating in the training will include fall-related concerns, health-related quality of life, balance performance, gait, physical activity, muscle strength in lower extremities, number of falls and compliance with training.

**Discussion:**

This study will generate new understanding of effective strategies for transferring research to clinical practice and thereby reduce an important knowledge gap, as well as aid decision-making for future implementation of evidence-based methods. Furthermore, it will contribute to improved balance and gait, increased level of physical activity and function, and improved health-related quality of life for the individuals participating in the programme.

**Trial registration:**

ClinicalTrials.govNCT02909374. Registered on September 21, 2016

## Background

Balance training and physical activity have been found to have positive effects on fall prevention, balance control and fear of falling [1–3]. However, despite convincing evidence on the efficacy and effectiveness of balance training and physical activity, there is a gap between research findings and what is performed and provided in community-based and clinical settings. Efficacy is the impact in studies carried out under ideal, researcher-controlled circumstances, whereas effectiveness is the impact when study conditions resemble real-world clinical practice [4].

The literature features many studies reporting on the effectiveness of certain interventions (treatments, methods and training programmes), many of which may have taken years for the researchers to develop. However, few of the researchers take their results further into clinical practice, and if they do, it may take years for them to be translated into daily use. This research-practice lag means that possible health gains are not achieved as quickly as would be desirable, resulting in a considerable knowing-doing gap, i.e. a gap between what is known from research and what is consistently delivered in practice [5]. Interventions with proven effectiveness should therefore be transferred into routine clinical practice and studied under more realistic conditions as soon as possible, with the evaluation of the implementation being an integral part of the studies. The purpose of this paper is to describe the design of a cluster-randomized implementation study carried out in primary health care setting evaluated with a mixed-method approach.

Our aim is to evaluate the effectiveness of two different strategies to facilitate the implementation of an intervention, the StayBalanced balance training programme, in primary health care, including evaluation of relative changes and maintenance in patient outcomes between intervention arms over 24 months.

## Methods/design

This project is designed as a hybrid type 3 trial [6]. Accordingly, the primary aim is to investigate the effectiveness of two different strategies to facilitate the implementation of the StayBalanced training programme, and the secondary aim is to assess patient outcomes of the programme in the form of number of falls, fall-related concerns, balance performance, health-related quality of life, gait, muscle strength and physical activity. The project is a two-arm cluster-randomized study with a mixed-method approach, carried out in clinical primary health care settings.

The different components of the project and how they will be evaluated are described below.

### The intervention: StayBalanced training programme

The StayBalanced training programme was designed and developed based on well-established principles of exercise and on the knowledge that balance control relies on the interaction of several physiological systems, as well as interaction with environmental factors and the performed task [7]. The programme includes exercise with dual- and multi-task performance, i.e. performance when a person’s attention is divided between a motor task and a cognitive task, a natural component of daily activities that may increase the risk of falling in older adults. Since dual tasking has a major impact on daily physical performance, dual- and multi-task exercises are important parts of the balance training programme [8].

As training adaptions are specific for the system trained, this programme follows the principle of specificity in that it is based on exercises targeting various systems for postural control aiming to improve balance performance in specific situations that can occur in daily life, such as regaining postural stability after a perturbation or being able to suddenly avoid an obstacle, with retained balance, while simultaneously walking and answering a question. Furthermore, it is progressive because the exercises can be performed at different levels making it progressively challenging for each individual throughout the programme. The training is conducted as an individually tailored group programme [7]. The 12-week balance training will be performed twice per week in 1-h sessions. The training will be led by physiotherapists or trained leaders.

**Fig. 1:**
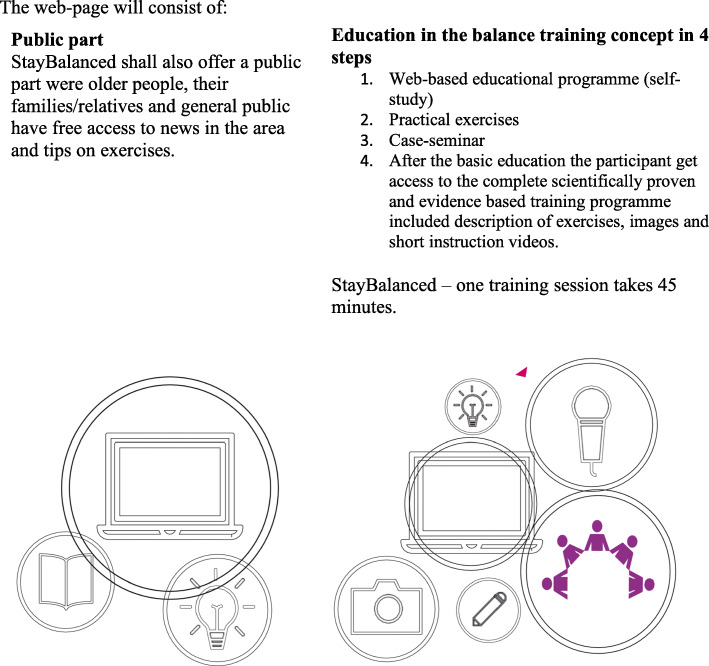
Overview of the content on the website, StayBalanced

**Fig. 2:**
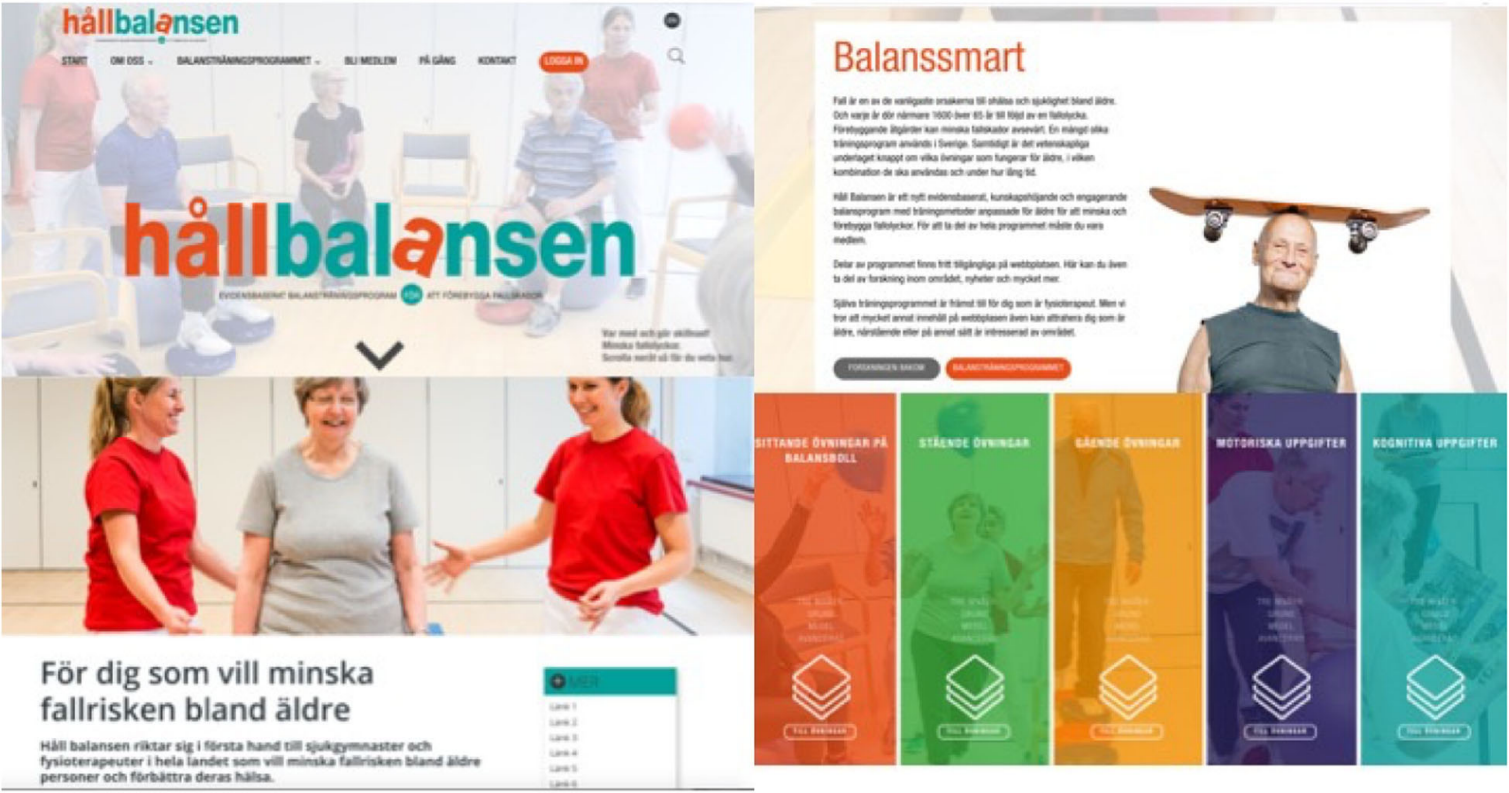
Examples from the Swedish website StayBalanced

The StayBalanced programme will be launched and distributed to clinical settings by an interactive website that will be innovative, useful, functional and easily available. The website will give physiotherapists access to the new evidence-based training programme and thereby help to prevent falls amongst older people. The StayBalanced programme provides practical tools, knowledge and information that facilitates different learning styles, creating awareness and attracting interest, and not least, steps to take active action to prevent falls amongst people over 65 years (see Figs. 1 and 2 for an overview of the content on the Swedish website).

### Patient outcomes

**Fig. 3:**
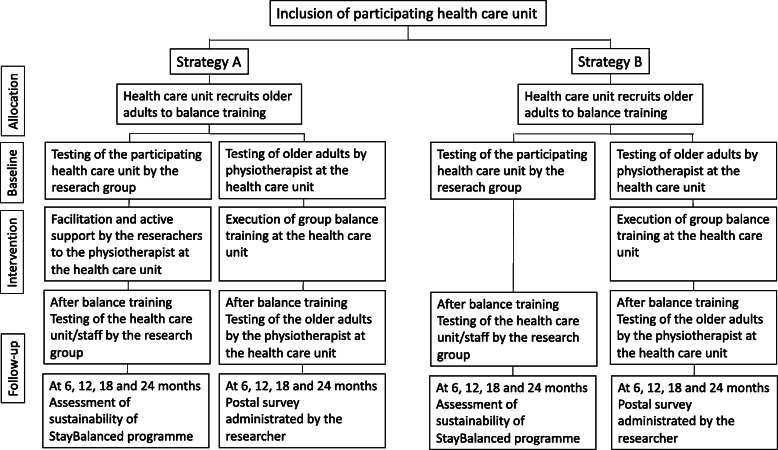
The project design for clinics and participants, showing start-up, baseline testing and follow-up

The assessments of patient outcomes will be performed at the primary health care unit by the regular physiotherapist before and after the training period, at 3 months (Table 1). All assessments are valid for older adults, i.e. the assessors will not be blinded for group allocation. Thereafter, the participants will be followed by the researchers up to 2 years after inclusion. During the follow-up period, at 6, 12, 18 and 24 months, the assessments will consist of a postal survey with a questionnaire regarding falls, consumption of health care, fall-related concerns, heath-related quality of life and self-reported physical activity, see Fig. 3. In addition, physical activity will be objectively assessed using accelerometers or pedometers, delivered by mail together with the questionnaire.

**Table 1:** Patient outcomes

Patient outcome	Assessment in the study	Instrument for assessment
Fall-related concerns	Assessed with questionnaires throughout the study period, from baseline to 2 years	Concerns about falling (Falls Efficacy Scale-International) [9] Fear of falling (single-item question “Are you afraid of falling?”) [10]
Number of falls	Assessed before and after the balance training period by the regular physiotherapist	“Please indicate by a number how many falls you have experienced during the last year/since the baseline assessment?”
Compliance with training and intervention protocol	Assessed during the balance training period by the regular physiotherapist	Checklist—attendance at training session and adherence to the intervention protocol
Health-related quality of life	Assessed with questionnaires throughout the study period, from baseline to 2 years	EQ 5D [11]
Physical activity	Assessed with questionnaires and pedometers and accelerometers throughout the study period, from baseline to 2 years	Pedometers or accelerometers Frändin-Grimby Activity Scale [12]
Balance performance	Assessed before and after the balance training period by the regular physiotherapist	MiniBESTest [13] or Bergs Balance Scale [14]
Gait	Assessed before and after the balance training period by the regular physiotherapist	10-m walk test [15]
Muscle strength	Assessed before and after the balance training period by the regular physiotherapist	The 30-s chair stand test [16]

### Implementation strategies

The project compares two strategies to achieve favourable implementation outcomes. Implementation strategy A involves an intervention including facilitation, whereby the researchers support the implementation process with a concerted educational effort at the introduction of the programme, in addition to the web-based education about how to run the programme, which exercise to begin with, what should be considered about security, etc. We will also follow the trainers closely by taking part in the training sessions as observers and facilitators to ensure that the training adheres to the intended progression and is challenging enough for the participants.

Implementation strategy B will consist simply of a web-based introduction of the programme, an overview on how to run the balance training, what should be considered about security, etc., and the trainers will then be followed by observers.

Strategy A is more resource-demanding than strategy B, i.e. being more akin to an intervention provided under efficacy-oriented conditions, whereas strategy B may be considered in terms of being more effectiveness-oriented.

### Implementation determinants and outcomes

The project applies the Consolidated Framework for Implementation Research framework [17] describing a number of determinant domains that have been found to mediate the impact of implementation strategies on implementation outcomes. Research in implementation science has established that successful implementation of new methods in health care settings is influenced by an interplay between four types of determinants: (I) the characteristics of the implementation object (intervention, practice, routine, programme, etc.), (II) the implementers (typically health care professionals), (III) the target population (usually patients) and (IV) the context of the implementation [17, 18]. To assess the implementation determinants, data will be collected by means of (1) interviews with health care professionals and older adults (individually or in focus groups), (2) observations during training sessions at the different settings and (3) questionnaires regarding demographic data of the implementers, older adults and settings. Table 2 presents more detailed information about the different implementation determinants.

**Table 2:** Implementation determinants that mediate between the implementation strategies and implementation outcomes

Implementation determinant	Explanation	Applied in this project	Data collection
Characteristics of the implemented “object”	Features of the practices (programme, service, methods, etc.) that are being implemented, e.g. knowledge-based or evidence-based practices that have been found to be effective in the research.	In this project, the implementation “object” is the StayBalanced programme, which is described in detail in a scientific publication and launched to clinicians by a web page.	Questionnaire answered by the professionals; interviews with the professionals
Characteristics of the target population	Features of the receivers of the implemented practice, i.e. the ultimate target population, usually patients.	Investigate the features of the target populations (older adults) who seek care due to balance deficits and fear of falling.	Interviews with the participating older adults
Characteristics of the implementers	Features of the professionals who use and deliver the practice. These implementers are those responsible for delivering the practice.	Features of the professionals who deliver the programme; investigate the features of the implementers, the professionals who deliver the programme.	Interviews with the professionals and questionnaires answered by the professionals
Characteristics of the context in which the implementation occurs	The context is the social environment in which implementation takes place; the context represents influences on the implementation process and impact that is, at least partially, beyond the control of the implementers and target population.	Investigate the features of the context in the clinical settings.	Checklists fulfilled by the professionals and researchers; interviews with the professionals and managers in the clinical settings; observations

The impact of the implementation strategy (A or B) on the implementation outcomes is mediated by four implementation determinants. In this project, four types of implementation outcomes will be investigated, as shown in Table 3. Data will be collected with interviews with professionals (individually or in focus groups), by questionnaire, the evidence-based practice attitude scale [19] and observations of training sessions at the different settings; long-term follow-up will be done by telephone interviews.

**Table 3:** Implementation outcomes

Implementation outcome	Applied in this project	Data collection
Acceptability	The professionals’ and older adults’ attitudes towards the balance programme and to what extent the professionals and older adults accept the programme	Questionnaire, evidence-based practice attitude scale [19]; interviews with the professionals
Feasibility	Description of how the balance programme fits into regular clinical practice	Interviews with the professionals
Fidelity	The professionals’ fidelity to the training protocol, which changes had to be done to conduct the program and do they still follow the theoretical background for conducting progressive balance training	Interviews with the professionals and observation of the professionals
Sustainability	The operation of the programme in clinical practice after 1 year	Telephone interviews with and questionnaires from the professionals

### Recruitment of participating units and participants

Participating clinics will be recruited strategically from different municipalities within Stockholm County, Sweden, i.e. from different sociodemographic areas, city and suburbs, to catchment area and population structure to achieve diverse participation.

Allocation will follow a computer-based randomization schedule that is sequentially numbered in blocks of four, using online software (https://www.randomizer.org/). This schedule will be generated by a research assistant. The included clinics will receive their allocation according to the randomization schedule in the same order they reported interest of participating in the study.

We plan to involve 20 primary health clinics, which will be allocated to two different implementation strategies. We estimate that 20 clinics will give enough power to explore which implementation strategy is more effective.

Since the outcomes for this implementation study are based on mixed methods (qualitative and quantitative data), we are not able to perform a power calculation to justify the number of recruited clinics. However, we estimate that 20 clinics will give enough data to explore which implementation strategy is more effective.

A power calculation was performed for the effectiveness part of the study, i.e. patient outcomes. The power calculation was based on MiniBESTest [13] and values from previous studies and our pilot study, resulting in a sample size of 16 participants at each primary setting to detect a 4-point difference (clinically relevant difference), assuming a mean variance or standard deviation (SD) of 3.6.

Participants in the balance training will be recruited by the staff in the present primary care facility in the vicinity of the clinics involved and by means of advertisement in local papers in the communities. Persons reporting their interest to take part in the study will be contacted by telephone for detailed information about the project and checked for eligibility by the staff at the clinic. The following are the inclusion criteria: 65 years or older, independent ambulatory indoor, self-perceived balance problems and/or fear of falling. The following are the exclusion criteria: fall-related fracture during the last 6 months, severely decreased vision, other diseases or constraints that might interfere with participation in the training programme. Eligible persons will be called for baseline testing at the primary health care unit (Fig. 3). There will be no special criteria for discontinuing or modifying allocated interventions.

**Fig. 4:**
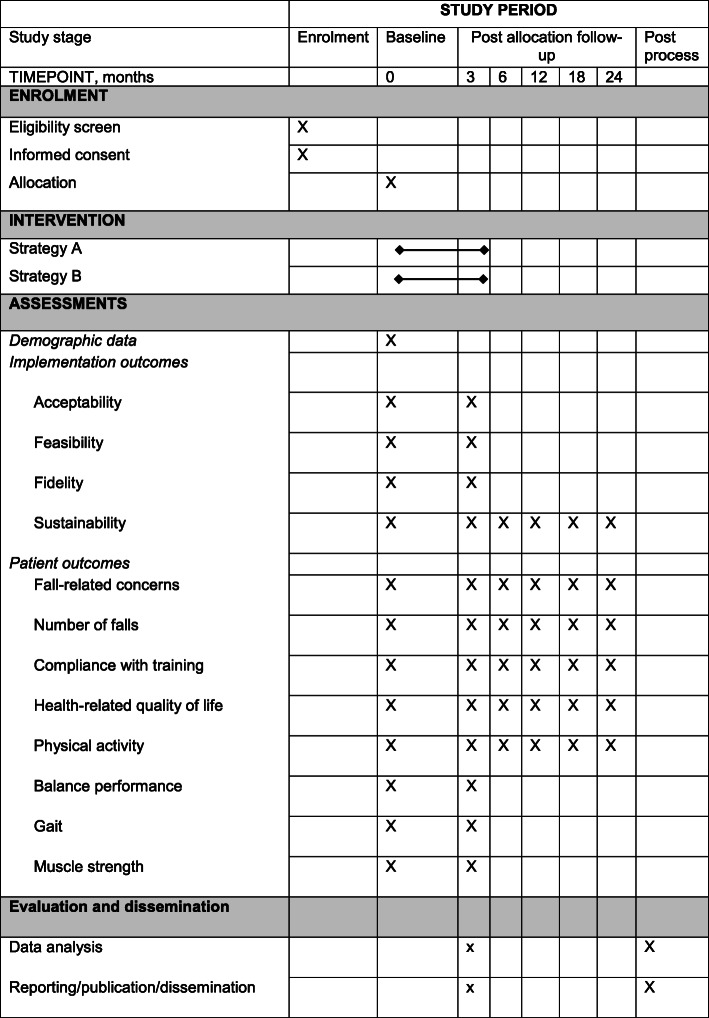
Study schedule (SPIRIT diagram of trial stages of enrolment, intervention, outcomes assessments and evaluation)

For details about the study schedule, see the SPIRIT diagram (Fig. 4).

### Data analysis and statistics

The implementation components will be evaluated with a mixed-methods approach using both quantitative and qualitative methods. Individual interviews and focus group discussions will be audiotaped, transcribed and encoded. For individual interviews, a semi-structured guide will be developed, and for focus groups, a guide with discussion themes will be used [20]. The final number of interviews and focus groups will be determined depending on the enrichment of the data. Qualitative data will be analysed using qualitative content analysis or phenomenography, depending on the specific research question. Patient-related data and descriptive data will be presented as the mean, median, percentage, minimum, maximum, 95% confidence interval and standard deviation depending on the characteristics of the data. Questionnaires (ordinal data) will be analysed with non-parametric statistics. Normally distributed interval data will be analysed with parametric statistics. Initial analyses will provide descriptions of the baseline characteristics by primary care facility and by intervention arm. Statistical testing of differences in baseline characteristics is not necessary but will provide assurance that the randomization has not produced a grossly unbalanced assignment. Patient-related outcome data will be analysed per protocol. For patient-related outcome data, missing data will be handled according to the different outcome measures, in relation to the preferable method for handling missing data for the specific measurement. Baseline characteristics of those not providing follow-up data will be examined to assess whether these are non-informative missingness.

The collection and analysis of qualitative and quantitative data will be carried out separately, and the findings will be compared and consolidated at the interpretation phase. By combining quantitative results obtained from the measurements and questionnaires with qualitative results obtained from individual interviews and focus groups, we will be able to get a more complete and comprehensive evaluation of the implementation [21, 22]. Triangulation will be used to explore if outcomes provide similar (complementary) or different (contradictory) findings [21]. No additional statistical analyses will be performed.

The design is open-label with only data analysts of patient outcomes being blinded, so unblinding will not occur.

### Ethical considerations

The study has been approved by the local ethics committee in Stockholm, Sweden (D.nr: 2016/415-31). One of the researchers will give information about the study and collect informed consent from the participants. They will be informed that they can end their participation without giving a reason for the decision.

Due to the challenging nature of the present balance training programme, we are aware of the increased risks compared with usual balance training of older adults and will take special precautions by having two trainers per group of 6–8 persons. However, we estimate the risk as small in comparison with the benefits of the intervention.

All data collected will be recorded in a protocol, one for each patient. Participants will receive a PIN code to prevent data being linked to participants. All encoded material will be entered in a database. The passkey to this will be stored separately and locked in a fireproof safe. Permission to establish a temporary register will be obtained from the Data Inspectorate under the General Data Protection Regulation (GDPR).

The audio file (also encoded) and the transcript from interviews will be stored in a locked, fireproof safe. The project has been registered at ClinicalTrials.gov under the trial registration number NCT02909374.

## Discussion

This project focuses on older adults’ health and prevention of falls, which is an important area of research because the older population is increasing. The study will increase the understanding of which coaching strategy is the most effective in reducing the risk of falling and fall-related injuries and contribute to decreasing a knowledge gap. Knowledge of which implementation strategy is the most effective for transferring an evidence-based balance training programme from a research to clinical practice may aid decision-making for future implementation of evidence-based methods.

In the literature, there is robust evidence that balance training has a positive impact on fall incidence. Therefore, further research on this link should not be prioritized over studies investigating the dissemination and implementation of balance training programmes needed [1–3]. For this reason, the present project was designed as a hybrid trial type 3, meaning that the focus is on testing implementation strategies while observing and gathering data on the programme’s impact on relevant outcomes [6]. Previous research has shown that reduced fall incidence and fear of falling has an impact on society as a whole, with lower costs linked to hospitalization, treatments and independency (society outcomes). However, more researcher is needed in the area of implementation of evidence-based interventions, underscoring the relevance of the design of the present study.

The project compares two implementation strategies, with the high-burden strategy A being more efficacy-oriented as it depends on considerable support from the researchers, and the low-burden strategy B being more effectiveness-oriented, since it does not rely on the researchers’ assistance to the same extent. Strategy B is intended to resemble real-world conditions for StayBalanced if the programme is more widely spread. The goal of an intervention efficacy study is to evaluate the impact of an intervention under ideal conditions, while an intervention effectiveness study is aimed at determining whether an intervention works under ordinary routine practice circumstances [23]. Studies of implementation strategies (i.e. a form of intervention) can also be considered in terms of their focus on efficacy or effectiveness. However, implementation studies conducted under optimal conditions, with the strategy to facilitate the implementation of an evidence-based intervention, for example, requiring substantial investments or other resources, may provide limited knowledge concerning the possibility to disseminate and implement the intervention more widely.

The project contributes to relevant knowledge of the use of clinical tests to predict falls and balance disorders and methods to overcome these problems. Our evaluation methods and training programme are easy to adopt in everyday clinical practice and will, in the very near future, have the potential to reach many older adults. The programme provides community-dwelling older adults with an available form of exercise designed to improve balance and reduce the fear of falling and prevent future falls. Individuals participating in the programme will not only reduce their risk of falling but also gain other health benefits related to active ageing, such as improved balance and gait, increased level of physical activity and function, and improved health-related quality of life.

### Trial implementation

The present study is approved by the head of the Department of Neurobiology, Care Sciences and Society at Karolinska Institutet, Stockholm, Sweden.

The Trial Steering Committee (TSC) consists of three responsible researchers, Alexandra Halvarsson, Ing-Mari Dohrn and Kirsti Skavberg Roaldsen. The members of TCS will meet every month over the course of the trial to oversee the conduct and progress. Moreover, we have a project group including research assistants, PhD student and the project manager. They are responsible for the day-to-day execution of the trial, i.e. recruitment, taking consent and conducting the trial. The trial will not have any stakeholders or public involvement group. However, funders of the trial will get a progress report yearly or at the end of the granted funding, depending on the funder’s guidelines.

Data Monitoring Committee was not considered as this was a low-risk intervention.

Implementing StayBalanced will not require alteration to usual care pathways (including use of any medication), and these will continue for both trial arms. There is no anticipated harm and compensation for trial participation.

Amendments to the intervention protocol will be noted by the physiotherapists and reported to the project group at the end of the training period.

## Data Availability

The datasets from the study will be available from the corresponding author on reasonable request. The obtained informed consent will be available from the corresponding author on request.

## Abbreviations

GDPR: General Data Protection Regulation
ALF: Regional Agreement on Medical Training and Clinical Research between Stockholm County Council and Karolinska Institutet
CFIR: Consolidated Framework for Implementation Research framework
